# Potential roles of oxidative distress on neurodegeneration in Parkinson's disease with neuropsychiatric symptoms

**DOI:** 10.3389/fnagi.2022.875059

**Published:** 2022-12-15

**Authors:** Dan-ning Li, Teng-hong Lian, Wei-Jiao Zhang, Ya-nan Zhang, Peng Guo, Hui-ying Guan, Jing-hui Li, Ming-yue He, Wen-jing Zhang, Wei-jia Zhang, Dong-mei Luo, Xiao-min Wang, Wei Zhang

**Affiliations:** ^1^Department of Neurology, Beijing Tiantan Hospital, Capital Medical University, Beijing, China; ^2^Center for Cognitive Neurology, Department of Neurology, Beijing Tiantan Hospital, Capital Medical University, Beijing, China; ^3^Department of Blood Transfusion, Beijing Tiantan Hospital, Capital Medical University, Beijing, China; ^4^Department of Physiology, Capital Medical University, Beijing, China; ^5^China National Clinical Research Center for Neurological Diseases, Beijing Tiantan Hospital, Capital Medical University, Beijing, China; ^6^Center of Parkinson's Disease, Beijing Institute for Brain Disorders, Beijing, China; ^7^Beijing Key Laboratory on Parkinson's Disease, Beijing, China

**Keywords:** Parkinson's disease, neuropsychiatric symptoms, free radicals, anti-oxidative enzyme, neuropathological proteins, cerebrospinal fluid

## Abstract

**Background:**

Neuropsychiatric symptoms (NPSs) belong to a category of non-motor symptoms of Parkinson's disease (PD), which seriously compromise the quality of life and prognosis of PD. This study focused on the correlations between NPSs, free radicals, neuroinflammatory factors, and neuropathological proteins in cerebrospinal fluid (CSF) in patients with PD, aiming to provide insights into the potential mechanisms and therapeutic target for PD with NPSs (PD-NPSs).

**Methods:**

In total, 129 patients with PD were enrolled and assessed by the Neuropsychiatric Symptoms Inventory (NPI); they were divided into the PD-NPSs group (75 patients) and PD with no NPSs (PD-nNPSs) group (54 patients). The levels of hydrogen peroxide (H_2_O_2_) and nitric oxide (NO), and hydroxyl radical (·OH), anti-oxidative enzyme, neuroinflammatory factors, and neuropathological proteins in CSF from patients with PD were measured. The levels of the above variables were compared between PD-NPSs and PD-nNPSs groups, and correlation analyses among the above variables were conducted.

**Results:**

(1) The levels of H_2_O_2_ and NO in CSF from the PD-NPSs group were significantly elevated compared with the PD-nNPSs group (*p* = 0.001), and NPI score positively correlated with the levels of H_2_O_2_ and NO (*r* = 0.283, *P* = 0.001; *r* = 0.231, *P* = 0.008). Reversely, total superoxide dismutase (tSOD) activity in CSF from the PD-NPSs group was significantly reduced compared with the PD-nNPSs group (*p* = 0.011), and negatively correlated with NPI score (*r* = −0.185, *p* = 0.036). (2) The tumor necrosis factor (TNF)-α level in CSF from the PD-NPSs group was significantly decreased compared with the PD-nNPSs group (*p* = 0.002) and negatively correlated with NPI score (*r* = −0.211, *p* = 0.016). (3) The total tau (T-tau) level in CSF from the PD-NPSs group was significantly higher than in the PD-nNPSs group (*p* = 0.014) and positively correlated with the NPI score (*r* = 0.167, *p* = 0.060). (4) The levels of H_2_O_2_ and NO positively correlated with the T-tau level in CSF from the PD-NPSs group (*r* = 0.183, *p* = 0.039; *r* = 0.251, *P* = 0.004), and the levels of TNF-α and T-tau showed a negative correlation (*r* = −0.163, *p* = 0.067).

**Conclusion:**

Oxidative distress characterized by the elevations of H_2_O_2_ and NO levels may closely correlate with the neurodegeneration in brain regions related to PD-NPSs. Thus, therapeutic antioxidants may become an important target for PD-NPSs therapy.

## Background

Parkinson's disease (PD) is a prevalent neurodegenerative disease. Apart from motor symptoms, patients with PD exhibit neuropsychiatric symptoms (NPSs), including depression, anxiety, apathy, euphoria, sleep disorders, appetite disorder, irritability, agitation, aberrant motor behavior, disinhibition, hallucination, and delusion. It was found that NPSs existed even before the onset of motor symptoms and deteriorated with PD progression (Aarsland and Kramberger, 2015). At the late stage of PD, NPSs compromised the life quality of patients more than motor symptoms did (Mueller et al., 2018) and brought heavy burdens to their families. Thus, early recognition and intervention of NPSs are of great significance for patients with PD.

Pathologically, PD is characterized by Lewy bodies, the main component of which is aggregated, mutated, and abnormally modified α-synuclein. Lewy bodies deposit in the substantia nigra and other brain regions, leading to neuronal degeneration and death through a variety of mechanisms. In addition to α-synuclein, there were also Alzheimer's disease (AD)-like pathological changes in the brains of patients with PD, including β amyloid (Aβ)_1 − 42_ and phosphorylated tau (P-tau) (Compta and Revesz, 2021), the main components of neuroinflammatory plaques and neurofibrillary tangles in the brains of patients with AD, respectively. There were few studies focusing on the correlation between NPSs and neuropathological proteins in patients with PD. Our previous studies showed that the α-synuclein level in CSF from PD patients with apathy was significantly increased compared to those with no apathy, and there was a significantly positive correlation between the score of the apathy scale and α-synuclein level in CSF (Wang et al., 2016). However, the correlation between the overall NPSs and neuropathological proteins in CSF from patients with PD has not been reported yet.

Among the multiple mechanisms relating to PD, oxidative distress and neuroinflammation play important roles in neuronal degeneration and death. However, most studies have focused on their effect on the motor symptoms of PD. As for non-motor symptoms of PD, NPSs were found to be related to the degenerated neurons and dysregulated neurotransmitters in certain specific brain regions. The correlations of depression with the variables of oxidative distress and neuroinflammation in CSF from patients with PD have been widely studied. Neuroinflammation plays a crucial role in both depression and PD, and it was reported that activated microglia and astrocytes in PD secreted multiple cytokines and interfered with physiological processes to induce depression. Conversely, in patients with depression, neuroinflammation and oxidative distress caused neurodegeneration, leading to PD (Tran et al., 2021). Apart from depression, other NPSs, such as anxiety and fatigue also correlated with neuroinflammatory factors in CSF of patients with PD (Lindqvist et al., 2013). However, the correlations of neuropathological proteins with the levels of oxidative distress and neuroinflammation in CSF from PD with patients with NPSs (PD-NPSs) has not yet been addressed.

Oxidative distress is characterized by the body's robust production of free radicals, including hydrogen peroxide (H_2_O_2_), nitric oxide (NO), hydroxyl radical (·OH), and the prominent reduction of the anti-oxidative enzyme, the total superoxide dismutase (tSOD). H_2_O_2_ and NO cause damage to the cell structure in many ways. They act as a second messenger in activating downstream signal pathways, including physiological and cytotoxic pathways. ·OH is highly oxidative and leads to cellular damage. We previously found that the levels of ·OH and H_2_O_2_ in CSF were significantly elevated in PD patients with apathy compared to those with no apathy (Wang et al., 2016). Moreover, apathy scale score positively correlated with the levels of ·OH and H_2_O_2_ in CSF, and H_2_O_2_ level significantly and positively correlated with α-synuclein level in CSF from PD patients with apathy, suggesting that excessively elevated α-synuclein in the brain might be related to PD with apathy through oxidative distress (Wang et al., 2016).

Neuroinflammation is characterized by the excessive activation of microglia and overproduction of neuroinflammatory factors, which participate in the pathogenesis of PD. For example, it was shown that the levels of interleukin (IL)-1 and tumor necrosis factor (TNF) -α in substantia nigra and serum from patients with PD were significantly increased, which activated downstream signaling pathways and led to neurodegeneration through the mechanisms of mitochondrial dysfunction and apoptosis (Liddelow et al., 2017). There is currently no study investigating the relationship between neuroinflammation and NPSs in patients with PD.

Based on the current research stated above, we assumed that the neuropathological proteins might cause oxidative distress and neuroinflammation, leading to neuronal degeneration and death in the brain regions relevant to NPSs in patients with PD. The degenerated and dead neurons might release contents, such as neuropathological proteins inside neurons, further aggravating oxidative distress and neuroinflammation and eliciting severer neuronal degeneration and death. A vicious cycle among neuropathological proteins, oxidative distress and neuroinflammation, and neurodegeneration might be formed, causing progressive damage to the brain regions relevant to NPSs and leading to PD-NPSs.

In this study, demographic variables of patients with PD were collected, and a host of professional rating scales were used to evaluate NPSs, disease severity, and motor symptoms. The levels of free radicals, including NO, H_2_O_2_, and ·OH, neuroinflammatory factors, including IL-1β, IL-6, TNF-α, prostaglandin (PG) E_2_ and interferon (INF)-γ, neuropathological proteins, including α-synuclein, Aβ_1 − 42_, P-tau (T181), P-tau (S199), P-tau (T231), P-tau (S396) and total tau (T-tau), and the activity of the anti-oxidative enzyme of tSOD in CSF were measured. The above variables were compared between PD-NPSs and PD with no NPSs (PD-nNPSs) groups, and correlation analyses of the above variables were conducted in the PD-NPSs group.

## Methods

### Ethics statement

This study was approved by the Review Board of Beijing Tiantan Hospital, Capital Medical University, and written informed consent was obtained from all participants and their family members.

### Participants

Patients were diagnosed with PD according to the International Parkinson and Movement Disorder Society (MDS) Clinical Diagnostic Criteria for Parkinson's Disease (Postuma et al., 2015). A series of evaluations were conducted in order to include patients who met the criteria of clinically established PD or clinically probable PD. These evaluations included thorough inquiry of medical history, physical examination, levodopa test, and a variety of auxiliary examinations, such as the head-upright tilt test, electromyography of the anal sphincter, residual urine, brain magnetic resonance imaging, and brain positron emission tomography if conditions permitted.

Most of the patients with PD recruited in this study were in the early stage of the disease and did not take dopaminergic medications. Few patients were on regular medications for hypertension, type 2 diabetes, hyperlipidemia, etc., on their first visit. However, subjects on medications that have dopamine antagonist effects, block dopamine receptors (neuroleptics, such as phenothiazines), and deplete dopamine in striatum (reserpine, butyrophenones, metoclopramide, and prochlorperazine), anti-depressants, calcium channel blockers, and H1-type anti-histamines, etc., were carefully examined to distinguish drug-induced parkinsonism. Patients whose symptoms conformed to the characteristics stated below were considered as drug-induced parkinsonism: (1) Patients experienced a relatively subacute onset of PD-like symptoms after taking the aforementioned drugs; (2) PD-like symptoms presented symmetrically in the lower extremities with bradyinesia, rigidity, and gait instability, but little tremor; (3) PD-like symptoms listed above were reversible and took several months to disappear after drug withdrawal; and (4) Patients were not sensitive to dopaminergic therapy. Participants with other diseases might explain NPSs, such as stroke, intracranial infection, primary depression, and side effects of drugs; severe systematic diseases, such as heart failure, pulmonary diseases, gastrointestinal diseases, anemia, infectious diseases, chronic inflammatory diseases, and other conditions that might lead to poor compliance to the study were excluded.

According to the criteria stated above, 129 patients with PD were consecutively recruited from Beijing Tiantan Hospital, Capital Medical University.

### Procedures

In this study, 129 patients with PD were divided into PD-NPSs and PD-nNPSs groups according to the neuropsychiatric inventory (NPI) score. All patients were given a complete physical examination by certified clinicians. Demographic variables were collected, and clinical manifestations were comprehensively evaluated by using a body of rating scales. CSF samples were obtained from patients who signed informed consents, and the levels of free radicals, neuroinflammatory factors, neuropathological proteins, and the activity of the anti-oxidative enzyme of tSOD in CSF were measured.

### Assessments of clinical symptoms

#### NPSs

Clinically, the NPI is commonly used to evaluate NPSs. In PD, it comprehensively reflects a variety of NPSs of patients and is thus widely recommended for evaluating disease progression, therapeutic effect, and prognosis. NPI evaluates 12 NPSs, including depression, anxiety, apathy, euphoria, sleep disorders, appetite disorder, irritability, agitation, aberrant motor behavior, disinhibition, hallucination, and delusion. Patients were divided into PD-NPSs and PD-nNPSs groups when the NPI score was >0 point and 0 point, respectively.

The Hamilton depression (HAMD) scale for depression, Hamilton anxiety (HAMA) scale for anxiety, and Modified apathy estimate scale (MAES) for apathy were also assessed. The Pittsburgh sleep quality index (PSQI) and Epworth sleeping scale (ESS) were used to evaluate sleep disorders. Individual NPS score reflected by the related rating scale was always consistent with NPI score.

In this study, a very small percentage of patients with PD reached the extent to take anti-depressants, and they were closely monitored for side effects or drug-induced parkinsonism after being instructed to take the medicines.

### Disease severity

The disease severity of each PD patient was evaluated by Hoehn–Yahr (H–Y) stage, which classifies the severity of PD into stage 0, 1, 2, 3, 4, and 5.

### Motor symptoms

Motor symptoms of patients with PD were evaluated by the Unified Parkinson's Disease Rating Scale (UPDRS) III after overnight withdrawal from anti-PD drugs. In UPDRS III, items 20 and 21 evaluate tremor, item 22 evaluates rigidity, items 23–26 evaluate bradykinesia, and items 27–30 evaluate postural and gait abnormalities.

### Collections of CSF samples

Anti-PD drugs were withdrawn for 12–14 h before lumbar puncture if the patients' condition allowed, and longer time was considered unethical by our ethical committee. In total, 3 ml of CSF was obtained through the lumbar puncture and was obtained in a polypropylene tube between 7 and 10 a.m. under fasting conditions. CSF samples were immediately centrifuged at 4 °C at 3,000 rpm for 10 min in the laboratory. Each CSF sample was aliquoted and reserved for 0.5 ml per tube at −80°C, avoiding freezing and thawing in case of protein degradation.

### Measurements of H_2_O_2_, NO, ·OH, and anti-oxidative enzyme of tSOD in CSF

The levels of free radicals, including H_2_O_2_, NO and ·OH, in CSF from patients in PD-NPSs and PD-nNPSs groups were measured by using the chemical colorimetric method.

H_2_O_2_ reacts with molybdate acid to form a complex (Liu et al., 2013). NO meets oxygen and water to produce nitrate and nitrite, which react with nitrate chromogenic agent and produce red azo compounds (Goshi et al., 2019). Griess reagent reacts with ·OH to form a red substance (Singh and Hider, 1988). The amount of the above-colored materials can be calculated into the amount of respective free radicals through the chemical colorimetric method (Singh and Hider, 1988; Liu et al., 2013; Goshi et al., 2019). A064 kit, A018 kit, and A012 kit (Nanjing Jiancheng Biological Engineering Research Institute, Nanjing, China) were used for the measurements of H_2_O_2_, NO, and ·OH, respectively.

The activity of tSOD in CSF from patients in PD-NPSs and PD-nNPSs groups was measured by using the chemical colorimetric method (Loh et al., 2010). A001-3-2 kit (Nanjing Jiancheng Biological Engineering Research Institute, Nanjing, China) was used for the measurement.

### Measurements of neuroinflammatory factors in CSF

The levels of neuroinflammatory factors, including IL-1β, IL-6, TNF-α, PGE_2_, and INF-γ in CSF from patients in PD-NPSs and PD-nNPSs groups were measured by using an enzyme-linked immunosorbent assay (ELISA). 1R040 kit (Beijing DOP Biotechnology Co., Ltd, Beijing, China), 1R140 kit (RB Company, Shanghai, China), 1R350 kit (RapidBio Company, Shanghai, China), CSB-E07965h kit (CUSABIO Company, Wuhan, China), and 1R330 kit (RapidBio Company, Shanghai, China) were used for the measurements of IL-1β, IL-6, TNF-α, PGE_2_, and INF-γ, respectively.

### Measurements of neuropathological proteins in CSF

The levels of neuropathological proteins in CSF from patients in PD-NPSs and PD-nNPSs groups were measured by using ELISA. CSB-E18033h, CSB-E10684h, and CSBE12011h (CUSABIO Company, Wuhan, China) were used for the measurements of α-synuclein, Aβ_1 − 42_, and T-tau, respectively. KHB7031 kit, KHB7041 kit, KhB8051 kit, and KHO0631 kit (Invitrogen Company, Carlsbad, America) were used for the measurements of P-tau (T181), P-tau (T231), P-tau (S396), and P-tau (S199), respectively.

### Data analysis

Statistical analysis was performed by SPSS Statistics 25.0 (IBM Corporation, New York, USA). *P* value < 0.05 was considered statistically significant.

A Shapiro–Wilk W-test was used to test the normality distribution of continuous variables. Those which were normally distributed were presented as means ± standard deviations and were compared by the two-sample *t*-test. Those which were not normally distributed were presented as medians (quartiles) and were compared by a Mann–Whitney *U*-test. Discrete variables were compared by a Chi-square test.

Demographic information, as well as the levels of free radicals, neuroinflammatory factors, and neuropathological proteins in CSF were compared between PD-NPSs and PD-nNPSs groups.

Spearman correlation analyses were conducted between the levels of free radicals as well as neuroinflammatory factors, neuropathological proteins and NPI score in patients with PD.

Since disease stage might be a confounding factor, Spearman correlation analysis was performed between the H–Y stage and the levels of free radicals, neuroinflammatory factors, T-tau, and NPI score.

## Results

### Frequency of each NPS in patients with PD

Of the 129 patients with PD recruited in this study, 75 cases (58.1%) were in the PD-NPSs group and 54 cases (41.9%) were in the PD-nNPSs group.

A total of 12 NPSs were evaluated by NPI for all patients with PD. According to the frequency of NPSs from high to low, 48 cases (37.2%) were with depression, 47 cases (36.4%) with anxiety, 42 cases (32.5%) with apathy, 25 cases (19.4%) with sleep disorders, 23 cases (17.8%) with irritability, 10 cases (7.8%) with appetite disorders, eight cases (6.2%) with hallucination, five cases (3.9%) with aberrant motor behavior, five cases (3.9%) with disinhibition, five cases (3.9%) with delusion, and four cases (3.1%) with euphoria (Table 1).

**Table 1:** Frequency of each NPS in patients with PD.

	**Cases incidence**
NPS-total [cases/total, (%)]	75/129, 58.1%
NPS-1 delusion [cases/total, (%)]	5/129, 3.9%
NPS-2 hallucination [cases/total, (%)]	8/129, 6.2%
NPS-3 agitation [cases/total, (%)]	4/129, 3.1%
NPS-4 depression [cases/total, (%)]	48/129, 37.2%
NPS-5 anxiety [cases/total, (%)]	47/129, 36.4%
NPS-6 euphoria [cases/total, (%)]	4/129, 3.1%
NPS-7 apathy [cases/total, (%)]	42/129, 32.6%
NPS-8 disinhibition [cases/total, (%)]	5/129, 3.9%
NPS-9 irritability [cases/total, (%)]	23/129, 17.8%
NPS-10 aberrant motor behavior [cases/total, (%)]	5/129, 3.9%
NPS-11 sleep disorders [cases/total, (%)]	25/129, 19.4%
NPS-12 appetite disorders [cases/total, (%)]	10/129, 7.8%

The frequency of each NPS in patients with PD was evaluated by using proportion and percentiles.

### Demographic variables of PD-NPSs and PD-nNPSs groups

Demographic variables, including gender, age, age of onset, duration of disease, educational level, and levodopa equivalent daily dose (LEDD), were compared between PD-NPSs and PD-nNPSs groups. The results showed no significant differences in the above variables between the two groups (*P* > 0.05) (Table 2).

**Table 2:** Comparisons of demographic variables between PD-NPSs and PD-nNPSs groups.

	**PD-nNPSs group (*n* = 54)**	**PD-NPSs group (*n* = 75)**	***P***
Male/total [(cases/total), %]	33/54, 61.1%	39/75, 52.0%	0.304
Age (years, mean ± SD)	60.2 ± 11.0	58.4 ± 9.5	0.305
Age of onset (years, mean ± SD)	57.1 ± 10.9	54.6 ± 10.0	0.168
Disease duration [years, median (quartile)]	2.0 (1.4, 4.0)	3.0 (1.5, 5.0)	0.187
Education			0.256
Middle school and below	33/129 (25.6%)	53/129 (41.1%)	
High school and above	18/129 (16.3%)	22/129 (17.1%)	
H-Y stage [median (quartile)]	1.5 (1.0, 2.3)	2.0 (1.5, 2.5)	0.030^*^
UPDRSIII scores[median (quartile)]	16.5 (11.25, 28.5)	27.0 (17.0, 39.0)	0.000^**^
Levodopa equivalent dose [mg, median (quartile)]	0.0 (0.0, 356.25)	150.0 (0.0, 600.0)	0.228

* P < 0.05,

** P < 0.01.

Demographic variables were compared between PD-NPSs and PD-nNPSs groups. The distributions of gender and education were compared by using the chi-square test, the distributions of age and age of onset were compared by using two independent sample *t*-test, and the distributions of disease duration, H–Y stage, UPDRS III score, and levodopa equivalent dose were compared by using a Mann–Whitney *U*-test.

### Disease severity and motor symptoms in PD-NPSs and PD-nNPSs groups

Disease severity reflected by the H–Y stage was compared between PD-NPSs and PD-nNPSs groups. The data presented that the H–Y stage in the PD-NPSs group was significantly more advanced than that in the PD-nNPSs group (*P* = 0.000), indicating that the PD-NPSs group had more advanced disease progression than the PD-nNPSs group (Table 2).

Motor symptoms rated by the UPDRS III scale were compared between PD-NPSs and PD-nNPSs groups. The data displayed that the score of UPDRS III in the PD-NPSs group was significantly higher than that in the PD-nNPSs group (*P* = 0.030), implying that the PD-NPSs group had more severe motor symptoms than the PD-nNPSs group (Table 2). To exclude the possible confounding effect of the disease stage, related correlation has been conducted and no significant correlations between the H–Y stage and the levels of free radicals, neuroinflammatory factors, and T-tau in CSF were found. Consequently, disease stage was not a confounding factor of the levels of H_2_O_2_, NO, and ·OH, neuroinflammatory factors, and T-tau in CSF.

### Comparisons of free radicals and anti-oxidative enzyme of tSOD in CSF between PD-NPSs and PD-nNPSs groups

**Figure 1:**
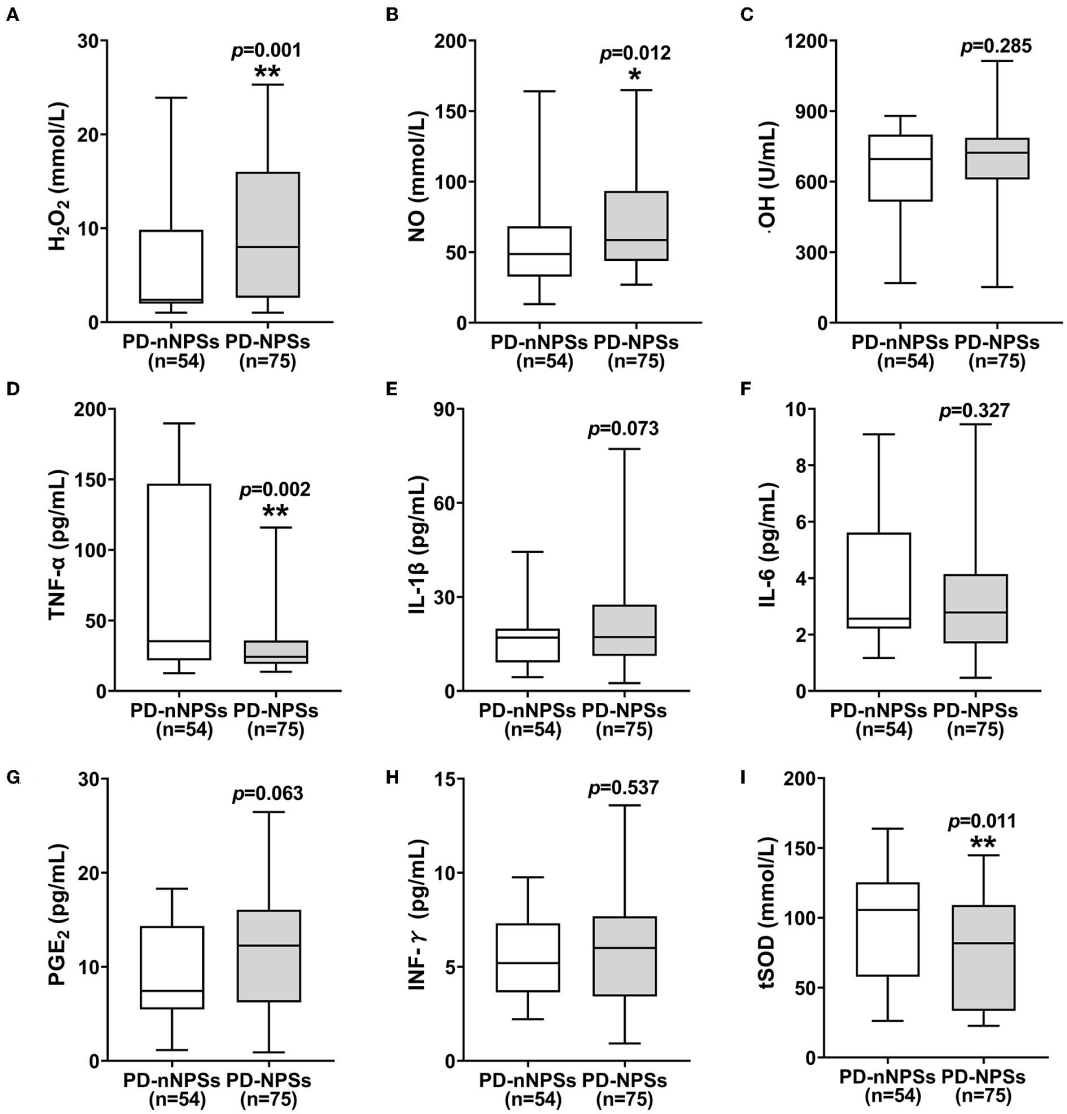
**(A–I)** The levels of free radicals and neuroinflammatory factors and the activity of tSOD in CSF were compared between PD-NPSs group and PD-nNPSs group. The results suggested that the levels of H_2_O_2_ and NO in CSF were significantly elevated, and the activity of tSOD was significantly reduced in CSF from PD-NPSs group compared to that from PD-nNPSs group.

**Figure 2:**
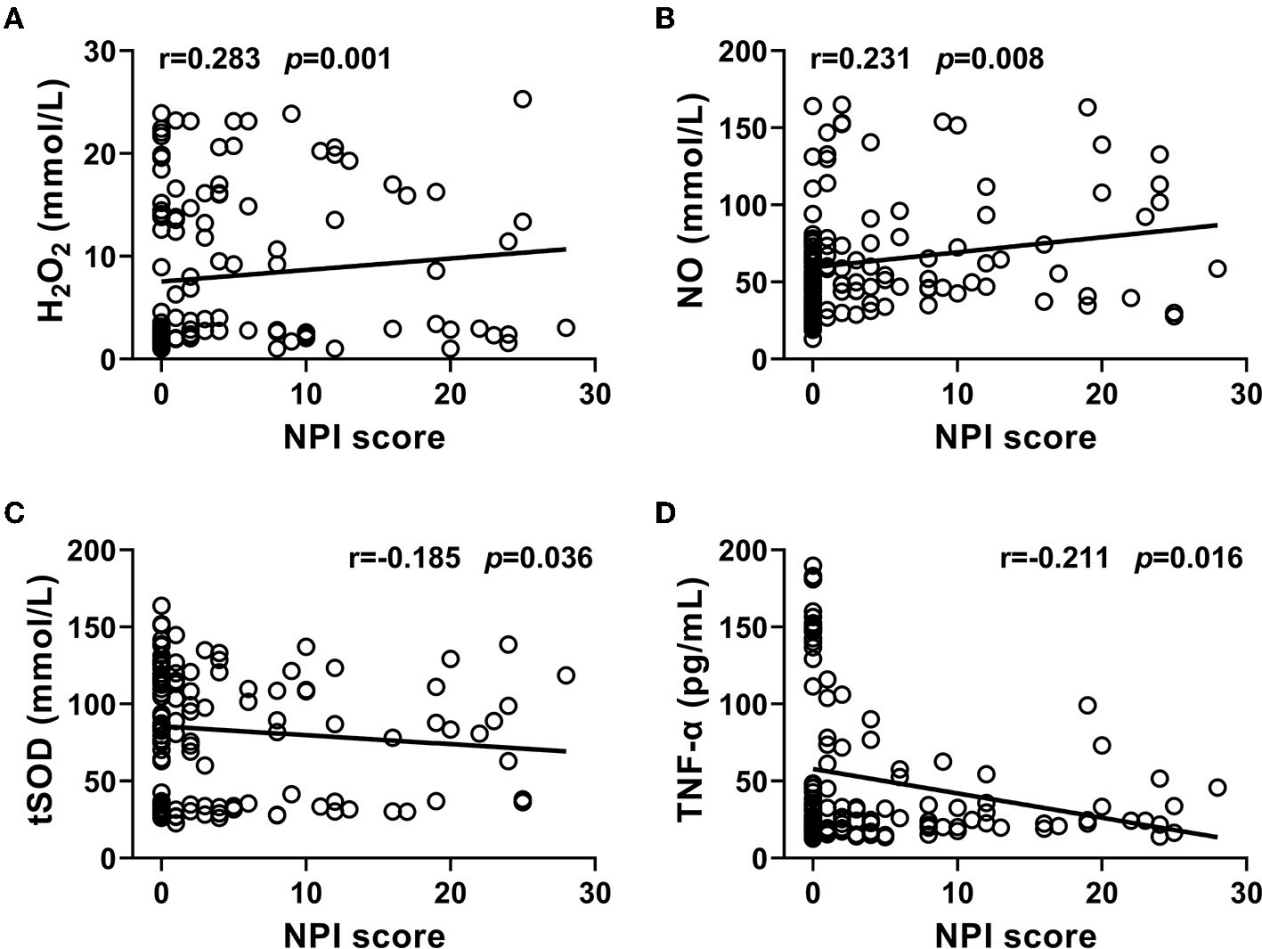
**(A–D)** Correlations of NPI score with the levels of H_2_O_2_, NO, and TNF-α, and the activity of tSOD in CSF from PD patients were analyzed. The results indicated that NPI score significantly and positively correlated with the levels of H_2_O_2_ and NO, and negatively correlated with the activity of tSOD and the level of TNF-α in CSF from PD patients.

In the free radicals measured, the levels of H_2_O_2_ and NO in CSF from the PD-NPSs group were significantly elevated compared with those from the PD-nNPSs group (*P* = 0.001, *P* = 0.012) (Table 3, Figures 1A,B). There was no difference in the ·OH level in CSF between the two groups (Table 3, Figure 1C). Further correlation analyses were performed between NPI score and the levels of H_2_O_2_ and NO in CSF from patients with PD. The results indicated that the NPI score significantly and positively correlated with the levels of H_2_O_2_ (*r* = 0.283, *P* = 0.001) and NO (*r* = 0.231, *P* = 0.008) in CSF (Table 4, Figures 2A,B).

**Table 3:** Comparisons of the levels of free radicals and neuroinflammatory factors, and the activity of tSOD in CSF between PD-NPSs and PD-nNPSs groups.

	**PD-nNPSs group (*n* = 54)**	**PD-NPSs group (*n* = 75)**	***P***
H_2_O_2_ [mmol/L, median (quartile)]	2.4 (2.0, 9.8)	8.0 (2.6, 16.0)	0.001^**^
NO [mmol/L, median (quartile)]	48.6 (32.7, 68.3)	58.6 (43.7, 93.4)	0.012^*^
·OH [U/mL, median (quartile)]	691.5 (504.3, 796.5)	722.5 (609.1, 786.9)	0.285
TNF-α [pg/mL, median (quartile)]	35.3 (21.7, 147.0)	24.3 (19.2, 35.8)	0.002^**^
IL-1β [pg/mL, median (quartile)]	17.0 (9.1, 19.9)	17.2 (11.2, 27.5)	0.073
IL-6 [pg/mL, median (quartile)]	2.6 (2.2, 5.6)	2.8 (1.7, 4.1)	0.327
PGE_2_ [pg/mL, median (quartile)]	7.4 (5.5, 14.4)	12.2 (6.2, 16.1)	0.063
INF-γ [pg/mL, median (quartile)]	5.2 (3.6, 7.3)	6.0 (3.4, 7.7)	0.537
tSOD [U/mL, median (quartile)]	81.8 (33.4,109.2)	105.6 (57.8,125.4)	0.011^*^

* *P* < 0.05,

** *P* < 0.01.

Two independent sample *t*-tests were used to compare the levels of free radicals and neuroinflammatory factors between PD-NPSs and PD-nNPSs groups.

**Table 4:** Correlations of NPI score with the levels of free radicals and neuroinflammatory factors, and the activity of tSOD in CSF from patients with PD.

	***r***	***P***
H_2_O_2_	0.283	0.001^**^
NO	0.231	0.008^**^
TNF-α	−0.211	0.016^*^
tSOD	−0.185	0.036^*^

* P < 0.05,

** P < 0.01.

Spearman analysis was used to evaluate the correlations between the levels of free radicals and neuroinflammatory factors in CSF with NPI scores in patients with PD.

At the same time, it was found that the activity of tSOD, a pivotal anti-oxidative enzyme, in CSF from the PD-NPSs group was significantly reduced compared with that from the PD-nNPSs group (*P* = 0.011) (Table 3, Figure 1I). Further analysis suggested that the NPI score significantly and negatively correlated with the activity of tSOD (*r* = −0.185, *P* = 0.036) in CSF from patients with PD (Table 4, Figure 2C).

### Comparisons of neuroinflammatory factors in CSF between PD-NPSs and PD-nNPSs groups

In the neuroinflammatory factors measured, TNF-α level in CSF from the PD-NPSs group was significantly decreased compared with that from the PD-nNPSs group (*P*= 0.002). There were no significant differences in the levels of IL-1β, IL-6, PGE_2_, and INF-γ in CSF between the two groups (Table 3, Figures 1D–H). Further correlation analysis was performed between NPI score and TNF-α level in CSF from patients with PD. It was shown that the NPI score significantly and negatively correlated with TNF-α level (*r* = −0.211, *P* = 0.016) in CSF from patients with PD (Table 4, Figure 2D).

### Comparisons of the levels of neuropathological proteins in CSF between PD-NPSs and PD-nNPSs groups

**Figure 3:**
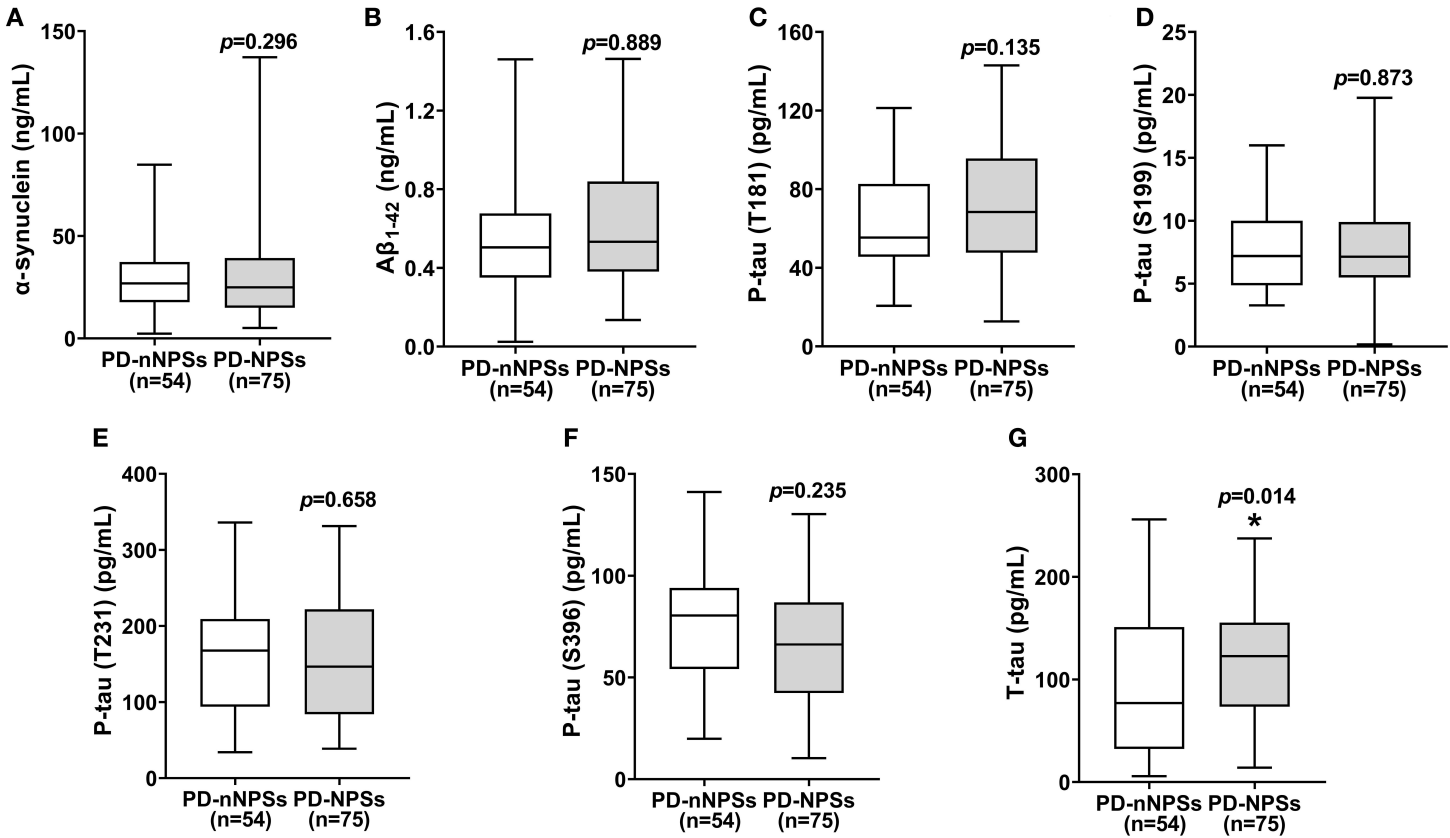
**(A–G)** The levels of neuropathological proteins in CSF were compared between PD-NPSs group and PD-nNPSs group. It was showed that T-tau level in CSF from PD-NPSs group was significantly higher than that from PD-nNPSs group.

**Figure 4:**
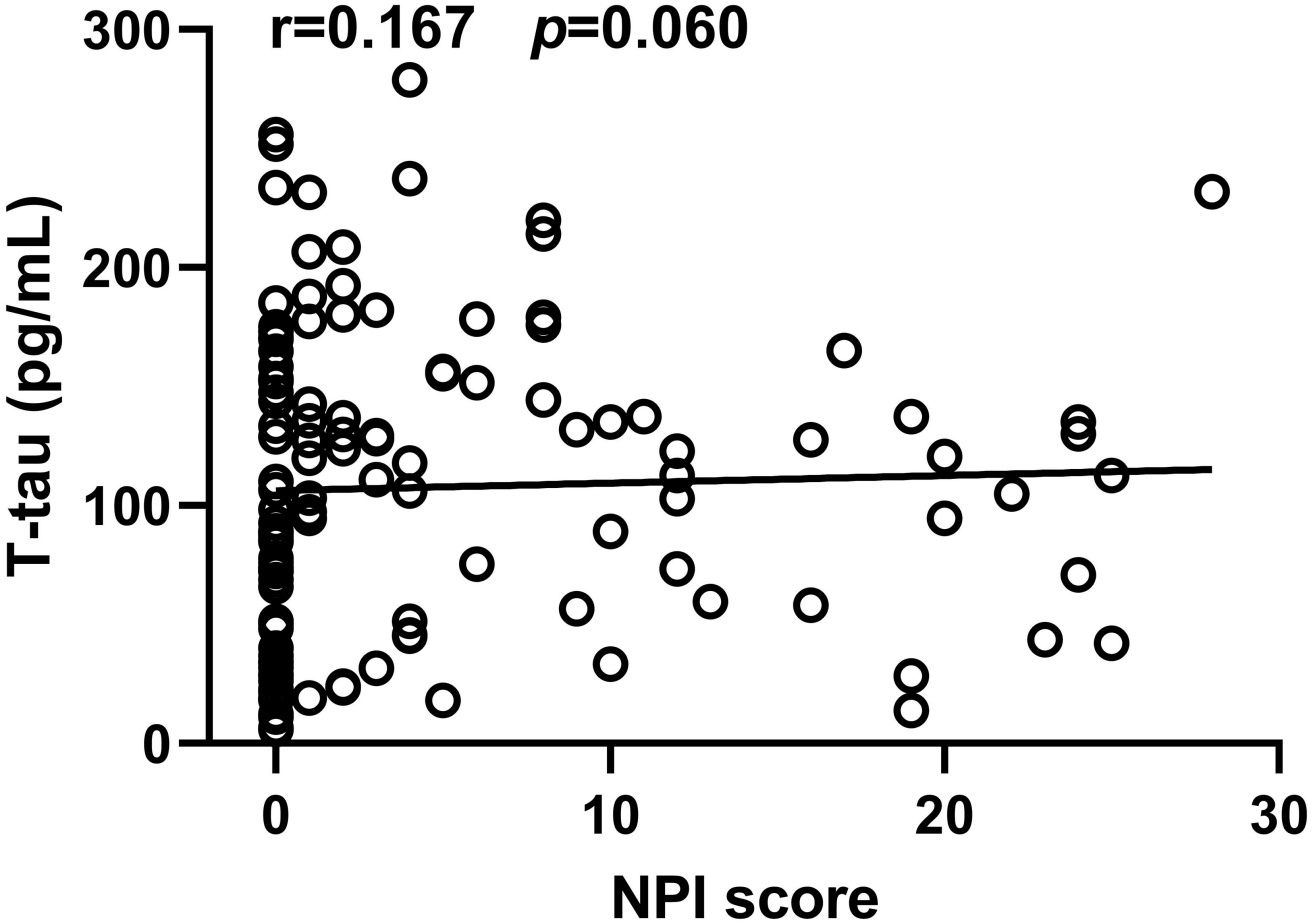
The correlation between NPI score and T-tau level in CSF from PD patients was analyzed. It was observed that NPI score significantly and positively correlated with T-tau level in CSF from PD patients.

In the neuropathological proteins measured, the T-tau level in CSF from the PD-NPSs group was significantly higher than that from the PD-nNPSs group (*P* = 0.014). There were no significant differences in the levels of α-synuclein, Aβ_1 − 42_, P-tau (T181), P-tau (S199), P-tau (T231), and P-tau (S396) between the two groups (Table 5, Figure 3). Furthermore, the correlation between NPI score and T-tau level in CSF from patients with PD was analyzed. The result presented that NPI score positively correlated with T-tau level in CSF from patients with PD (*r* = 0.167, *P* = 0.060) (Table 6, Figure 4).

**Table 5:** Comparisons of the levels of neuropathological proteins in CSF between PD-NPSs and PD-nNPSs groups.

	**PD-nNPSs group (*n* = 54)**	**PD-NPSs group (*n* = 75)**	***P***
α-synuclein [ng/mL, median (quartile)]	26.9 (17.7, 37.3)	25.0 (15.0, 39.2)	0.296
Aβ_1 − 42_ [ng/mL, median (quartile)]	0.5 (0.4, 0.7)	0.5 (0.4, 0.84)	0.889
P-tau (T181) [pg/mL, median (quartile)]	55.4 (45.5, 82.7)	68.4 (47.7, 95.6)	0.135
P-tau (S199) [pg/mL, median (quartile)]	7.2 (4.9, 10.0)	7.1 (5.5, 9.9)	0.873
P-tau (T231) [pg/mL, median (quartile)]	167.8 (94.0, 209.2)	146.6 (84.0, 222.0)	0.658
P-tau (S396) [pg/mL, median (quartile)]	80.4 (54.1, 94.0)	66.2 (42.3, 87.0)	0.235
T-tau [pg/mL, median (quartile)]	91.1 ± 66.7	119.3 ± 60.1	0.014^*^

* *P* < 0.05.

Two independent sample *t*-test was used to compare the levels of neuropathological proteins between PD-NPSs and PD-nNPSs groups.

**Table 6:** Correlation between the T-tau level in CSF and NPI score in patients with PD.

***R***	***P***
0.167	0.060

Spearman analysis was used to evaluate the correlations of the levels of free radicals and neuroinflammatory factors with the T-tau level in CSF in patients with PD.

### Correlations of the levels of free radicals and neuroinflammatory factors, and the activity of anti-oxidative enzyme of tSOD with the levels of neuropathological proteins in CSF from the PD-NPSs group

In this study, correlation analyses of the levels of free radicals and neuroinflammatory factors, and the activity of anti-oxidative enzyme of tSOD with the levels of neuropathological proteins in CSF from the PD-NPSs group were performed.

**Figure 5:**
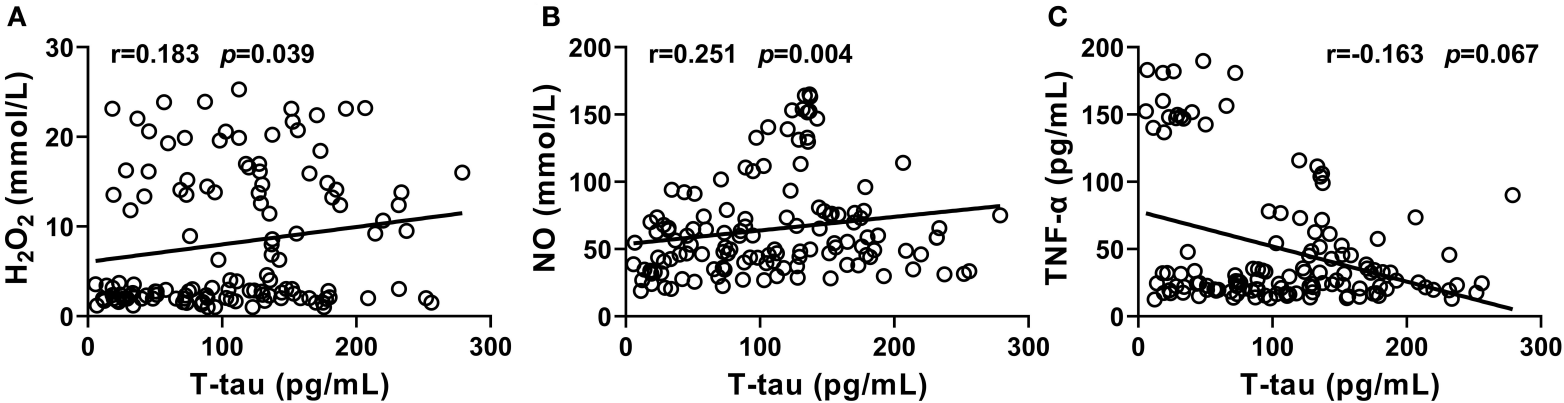
**(A–C)** Correlations of T-tau level with the levels of free radicals and neuroinflammatory factors, and the activity of tSOD in CSF from PD-NPSs group were analyzed. The results displayed that the levels of H_2_O_2_ and NO significantly and positively correlated with T-tau level in CSF from PD-NPSs group. The levels of TNF-α and T-tau in CSF showed a negative correlation with P value close to a significant point.

It was shown that the levels of H_2_O_2_ and NO significantly and positively correlated with T-tau level in CSF from the PD-NPSs group (*r* = 0.251, *P* = 0.004; *r* = 0.183, *P* = 0.039) (Table 7, Figures 5A,B). The levels of TNF-α and T-tau showed a negative correlation with a *P*-value close to a significant point (*r* = −0.163, *P* = 0.067) (Table 7, Figure 5C). However, tSOD activity and T- tau level in CSF from the PD-NPSs group showed no significant correlation (*r* = −0.112, *P* = 0.212).

**Table 7:** Correlations of the levels of free radicals and neuroinflammatory factors with neuropathological proteins in CSF from the PD-NPSs group.

	***r***	***P***
H_2_O_2_	0.183	0.039^*^
NO	0.251	0.004^**^
TNF-α	−0.163	0.067

* *P* < 0.05,

** *P* < 0.01.

Spearman analysis was used to evaluate the correlations of the levels of free radicals and neuroinflammatory factors with T-tau level in CSF in patients with PD.

## Discussion

### Frequency of each NPS of PD

Many patients with PD have more than one NPS; therefore, NPI was used to evaluate a series of NPSs for patients with PD recruited in this study.

Depression, anxiety, hallucination, and apathy are the most prevalent NPSs in patients with PD. Depression is characterized by low mood and lack of interest or pleasure and can be furthermore divided into major depression disorder, minor depression, and dysthymia according to the clinical manifestations. The prevalence of depression in patients with PD varied between 2.7 and 89.0% as a result of different populations recruited and diagnosis criteria adopted in different studies. Anxiety includes generalized anxiety disorder, panic disorder, agoraphobia, obsessive-compulsive disorder, social or specific phobias, and anxiety not otherwise specified. Generally, the incidence of anxiety ranges from 6 to 55%. The recent meta-analyses reported that the incidence of generalized anxiety disorder, social phobia, anxiety not otherwise specified, specific phobia and panic disorder were 14.0, 13.8, 13.3, 13.0, and 6.8%, respectively (Broen et al., 2016). Apathy is characterized by a lack of motivation and decreased goal-oriented behavior, and its incidence in patients with PD was between 17.0 and 60.0% (Bogart, 2011). In this study, the frequencies of depression, anxiety, and apathy in patients with PD were 37.2, 36.4, and 32.6% (Table 1), respectively, indicating that the above NPSs were very common in patients with PD.

Sleep disorders include abnormalities in sleep structure, breathing, movement, and behavior. The incidence of sleep disorders in patients with PD ranged from 50.0 to 81.0%. In this study, 19.4% of patients with PD had sleep disorders (Table 1), which might be related to the relatively early stage of disease (the median and quartile of the H–Y stage: 2.0 [1.0, 2.5]) of patients with PD recruited.

The frequency of hallucination is time-dependent and increases with disease progression. A cross-sectional study revealed that the incidence of visual hallucination ranged from 25.0 to 33.3%, while that of auditory hallucination was up to 20.0% (Fénelon and Alves, 2010). In this study, the frequency of hallucination in patients with PD was 6.2% (Table 1), lower than that reported by previous investigations, which might be related to the relatively short disease duration (the median and quartile of disease duration: 3.0 [1.5, 5.0] years) (Table 1) and early stage of disease (the median and quartile of the H–Y stage: 2.0 [1.0, 2.5]) (Table 1) of patients with PD recruited.

In addition to the above NPSs, the remaining NPSs of irritability, appetite and eating disorders, abnormal movement, disinhibition, delusion, euphoria, and agitation in patients with PD have been rarely investigated. In this study, the frequencies of these NPSs were 17.8, 7.8, 3.9, 3.9, 3.9, 3.9, 3.1, and 3.1% (Table 1), respectively.

### Demographic variables, disease severity, and motor symptoms in PD-NPSs and PD-nNPSs groups

Unlike findings reported by other investigators (Aarsland and Kramberger, 2015), results from this study showed no significant differences in gender, age, age of onset, disease duration, educational level, and LEDD between the two groups (Table 2), excluding the impacts of above demographic variables on PD-NPSs.

Previous studies focused on the relationship between a part of NPSs and motor symptoms in patients with PD. Results from a longitudinal study implied that depression contributed to the worsening of motor symptoms in patients with PD (Ng et al., 2015). In addition to depression, it was found that the more severe the anxiety and apathy, the more prominent the motor symptoms (Dujardin et al., 2014; Avanzino et al., 2018). Disease severity reflected by the H–Y stage was shown to be related to psychosis as well as depression in patients with PD. However, there has been no study on the correlations of overall NPSs with disease severity and motor symptoms in patients with PD. In this study, the PD-NPSs group had significantly advanced H-Y stage and elevated UPDRS III score compared with the PD-nNPSs group (Table 2), indicating that the disease was more severe and motor symptoms were more prominent in the PD-NPSs group than in the PD-nNPSs group. It was speculated that NPSs might potentially be involved with mechanisms similar to motor symptoms and disease progression of PD, underlying the development of neurodegeneration and depletions of multiple neurotransmitters, including dopamine, serotonin, noradrenaline, and acetylcholine (Nagy and Schrag, 2019). Therefore, NPSs are of great significance and should be comprehensively evaluated, early identified, and timely treated.

### Comparisons of free radicals and anti-oxidative enzyme of tSOD in CSF between PD-NPSs and PD-nNPSs groups

An abundant oxygen supply is essential to brain activity, and quantities of oxygen are converted into reactive oxygen species (ROS) through metabolism. It has been demonstrated that many physiological conditions, including excessive dopamine metabolism, higher levels of iron and calcium in substantia as well as mitochondrial dysfunction, can lead to severer oxidative distress. Oxidative distress results from the dysregulation of cellular redox activity, which is characterized by the imbalance between the productions of free radicals, including NO, H_2_O_2_, and ·OH, and the activities of endogenous antioxidant enzymes for removing free radicals, such as SOD, catalase, and glutathione peroxidase. The homeostasis of redox activity plays important roles in the host defense, gene transcription, regulation of synaptic plasticity, and apoptosis. It was found that when the levels of free radicals significantly exceeded the ability of antioxidants, signaling pathways relating to neuroinflammation and neuronal injury were elicited, which caused protein lysis, enzyme inactivation, lipid destruction, and deoxyribonucleic acid denaturation, induced damage to the neurons vulnerable to oxidative distress, and caused depletions of a variety of neurotransmitters, including dopamine, serotonin, and noradrenaline (Schieber and Chandel, 2014). It was demonstrated that neurons in the substantia nigra were particularly sensitive to the toxic effects of free radicals produced in the course of oxidative distress, and thus might be involved in the initiation and progression of motor symptoms of PD. Although there have been studies on the relationship between oxidative distress and PD (Hassanzadeh and Rahimmi, 2018; Musgrove et al., 2019), it is unclear whether oxidative distress is associated with NPSs in patients with PD. In this study, the levels of H_2_O_2_ and NO in CSF from the PD-NPSs group were significantly elevated compared with those from the PD-nNPSs group (Table 3), and the NPI score significantly and positively correlated with the levels of H_2_O_2_ and NO in CSF from patients with PD (Table 4). The above-mentioned results indicated that the elevated levels of H_2_O_2_ and NO might interrupt crucial pathways of downstream signaling transduction, compromise cell function, and thus contribute to NPSs. Hence, oxidative distress characterized by the robust generations of H_2_O_2_ and NO played an important role in the NPSs of PD.

Superoxide dismutase is a family of enzymes that catalyzes the dismutation of superoxide anions. SOD1, also named CuZn-SOD, contains copper and zinc and exists only in intracellular space. SOD2, also named Mn-SOD, contains Mn and exists in mitochondrial space. SOD3 contains copper and zinc and exists in extracellular spaces. SOD is crucial in the antioxidant physiological process, and several studies reported the altered activity of SOD in blood, CSF, and brain parenchyma in patients with PD (Tórsdóttir et al., 2006). SOD2 activity was increased in frontal cortex parenchyma in patients with PD (Ferrer et al., 2007), which reflected the oxidative damage of superoxide to brain parenchyma (Marttila et al., 1988). tSOD activity in CSF from patients with PD was decreased compared to that from health controls (Boll et al., 2008). SOD activity was pronouncedly increased in substantia nigra of patients with PD (Saggu et al., 1989). These findings from above-limited studies are inconsistent to some extent, thus no conclusion of the alteration of SOD activity in patients with PD has been drawn. Moreover, no study has investigated the alteration of SOD activity in patients with PD-NPSs. In this study, tSOD activity in CSF from the PD-NPSs group was significantly decreased compared with that from the PD-nNPSs group, and the NPI score significantly and negatively correlated with tSOD activity in CSF from patients with PD (Table 3). These data indicated that the excessive H_2_O_2_ and NO promoted tSOD production in brain parenchyma, thus decreasing its activity in CSF. Meanwhile, the higher level of H_2_O_2_ in CSF indicated that it was produced by tSOD, and then released and permeated into CSF, leading to the elevated level of H_2_O_2_ in CSF.

In conclusion, oxidative distress was very pivotal for PD-nNPSs due to the significant elevations of free radicals and altered distributions of anti-oxidative enzymes.

### Comparisons of neuroinflammatory factors in CSF between PD-NPSs and PD-nNPSs groups

In physical conditions, normal levels of neuroinflammatory factors are essential for a variety of cellular functions; however, excessively elevated neuroinflammatory factors are very harmful to neurons. Neuroinflammation was a key pathogenesis of PD, which was characterized by the over-activation of microglia in brain and robust productions of a series of neuroinflammatory factors, such as IL-1, IL-6, TNF-α, PGE_2_, and INF-γ (Pajares et al., 2020).

Neuroinflammation and oxidative distress are closely interrelated. In PD, on the one hand, oxidative distress promotes neuroinflammation, and neuroinflammation aggravates oxidative distress, resulting in a joint effort to accelerate neurodegeneration; on the other hand, neuronal death further propagates oxidative distress and neuroinflammation. Both oxidative distress and neuroinflammation activate a variety of signal pathways, cause morphological and functional damages to neurons, induce neuronal degeneration and death, and eventually lead to the development and progression of PD (Hassanzadeh and Rahimmi, 2018). There are investigations on the correlation between neuroinflammation and PD; however, whether neuroinflammation exerts a role in NPSs of PD is still unclear.

In this investigation, PD-NPSs were associated with oxidative distress; thus, it was speculated that PD-NPSs might also be associated with neuroinflammation, i.e., the levels of neuroinflammatory factors in CSF from the PD-NPSs group might be significantly increased compared to that from the PD-nNPSs group. However, the results showed that TNF-α level in CSF from the PD-NPSs group was significantly lower than that from the PD-nNPSs group, and NPI score significantly and negatively correlated with TNF-α level in CSF from patients with PD (Table 4). Meanwhile, the levels of other neuroinflammatory factors, including IL-1β, IL-6, PGE_2_, and INF-γ, were not significantly different between the two groups (Table 3), and did not correlate with NPI score in patients with PD (data not shown). It was previously reported that microglial activation induced the upregulation of proinflammatory enzymes and release of proinflammatory factors of TNF-α, indicating the role of TNF-α in neuroinflammation (Hassanzadeh and Rahimmi, 2018). However, contrary to our prediction in this study, TNF-α level in CSF from the PD-NPSs group was significantly decreased compared with that from the PD-nNPSs group, which might be because that most of the patients included were at the early stage of PD, and the decreased TNF-α implied the potential anti-inflammatory action but not proinflammatory effect on nNPSs at the early stage of PD. These results elucidate that, on the one hand, not all neuroinflammatory factors are always involved in each symptom of PD, and on the other hand, even when neuroinflammatory factors are involved, not all of them routinely play detrimental roles, and some may even exert a compensational effect on a certain symptom at the early stage of PD. Thus, neuroinflammatory factors may have more complex regulatory networks, and the exact influences of these factors on PD-NPSs need further investigation. Thus, data of this study indicate that oxidative distress, rather than neuroinflammation, plays a pivotal role in PD-NPSs. In future, we will include a control group to further elucidate the role of TNF-α on the pathogenesis of PD-NPSs.

### Comparisons of neuropathological proteins between PD-NPSs and PD-nNPSs groups

One of the pathological features of PD is the formation of Lewy bodies composed of pathologically aggregated, mutated, and abnormally modified α-synuclein. Lewy bodies are deposited within or beyond the substantia nigra and lead to neurodegeneration through a variety of mechanisms, causing motor and non-motor symptoms of PD. α-synuclein-containing Lewy bodies distribute in, monoaminergic, and cholinergic neurons in the brainstem at the early stage, and spreads to the limbic system and neocortex with disease progression. In addition, PD is also associated with the depositions of other neuropathological proteins, such as Aβ_1 − 42_ and P-tau, which are the major components of neuritic plaques and neurofibrillary tangles, the pathological features of AD, respectively. Aβ_1 − 42_ and P-tau were found to be associated with cognitive impairment in patients with PD (Yu et al., 2014; Schrag et al., 2017). However, there has been no study on the relationship between these neuropathological proteins and PD-NPSs.

It is well known that T-tau is a biomarker of neurodegeneration. The higher the level of T-tau, the more severe the degeneration of neurons and the lower the levels of related neurotransmitters. However, the role of T-tau in PD has limited investigations. Researchers found that the level of T-tau and its ratio to P-tau are related to cognitive function, but no studies have been conducted to elucidate its role on PD-NPSs. In this study, T-tau level in CSF from the PD-NPSs group was significantly higher than that from the PD-nNPSs group (Table 5), and NPI score positively correlated with T-tau level in CSF from patients with PD, in which *P*-value was near to statistically significant (Table 6), indicating that neurodegeneration in the PD-NPSs group was more remarkable than that in the PD-nNPSs. Since the correlation between T-tau level in CSF and NPI score did not reach a significant level, we will in the future measure the volume of specific brain regions relevant to NPSs by magnetic resonance imaging in addition to enhancing CSF samples from patients with PD, which may further validate the conclusion from this study.

α-synuclein is the major component of Lewy body. In this study, the measured forms of α-synuclein by the kit included soluble cytosolic monomer and oligomer. It was observed that the α-synuclein level in CSF not significantly correlated with PD-NPSs. Our previous investigations showed that not all of the symptoms of PD correlated with the α-synuclein level in CSF. For example, rapid eye movement sleep behavior disorder (RBD) (Hu et al., 2015), apathy (Wang et al., 2016), and fatigue (Zuo et al., 2016) correlated with the elevated α-synuclein level in CSF, while depression (Lian et al., 2020)and excessive daytime sleepiness (Hu et al., 2021) were not. For the first time, we found that oxidative distress, as reflected by robust elevations of H_2_O_2_ and NO in CSF, and subsequent neurodegeneration, as reflected by a significant elevation of T-tau in CSF, highly correlated with PD-NPSs. These data indicate that it is oxidative distress but not α-synuclein, that plays a key role in PD-NPSs. Thus, α-synuclein may directly correlate with part of the symptoms of PD through its neurotoxic effect on neurons in the related brain regions, or may not directly correlate with part of the symptoms of PD, but rather through other mechanisms, including iron deposition (Yu et al., 2013; Lian et al., 2019; Hu et al., 2021), neuroinflammation (Yu et al., 2013; Hu et al., 2021), oxidative distress (Wang et al., 2016), and decreased anti-oxidative activity, etc. The different roles and mechanisms of α-synuclein on individual symptoms of PD call for further investigations in the future.

There were no significant differences in the levels of Aβ_1 − 42_, P-tau (T181), P-tau (S199), P-tau (T231), and P-tau (S396) in CSF between PD-NPSs and PD-nNPSs groups. Thus, we speculated that PD-NPSs were associated with the degeneration of neurons, but not with PD-like or AD-like pathological changes.

### The relationships among PD-NPSs, oxidative distress, and neuropathological proteins

It was found that multiple NPSs in patients with PD were associated with the degeneration of serotoninergic, noradrenergic, and dopaminergic neurons and the subsequent dysfunctions of the relevant neurotransmitters in the brainstem. Depression and anxiety of PD were associated with the degeneration of serotoninergic neurons in raphe nucleus, noradrenergic neurons in locus coeruleus, as well as the degeneration of dopaminergic neurons and dysregulations of frontostriatal and mesocorticolimbic dopaminergic circuits (O'Callaghan et al., 2014; Castrioto et al., 2016). Autopsy results showed that the numbers of degenerated neurons in the substantia nigra of PD patients with depression were six times more than those without depression. Magnetic resonance imaging studies showed that PD patients with depression and anxiety had atrophy in the gray matter of the limbic system, reduction in white matter, and blood flow in the frontal lobe and anterior cingulate gyrus (Feldmann et al., 2008; Cardoso et al., 2009; Kostić et al., 2010; Surdhar et al., 2012; O'Callaghan et al., 2014; van Mierlo et al., 2015). PD with apathy was associated with greater dopaminergic denervation (Thobois et al., 2010) and gray matter atrophy in prefrontal, parietal and cingulate cortices, and nucleus accumbens (Reijnders et al., 2010; Carriere et al., 2014). It was found that PD with hallucination was related to high densities of Lewy bodies, as well as plaques and tangles in frontal, parietal and temporal lobes, amygdala and parahippocampus. Multiple neurotransmitters, including dopamine, serotonin, and acetylcholine were implicated in PD with hallucinations (de la Fuente-Fernández, 2013; Jacobson et al., 2014). Disturbed sleep in patients with PD was related to α-synuclein deposition in the locus coeruleus and raphe nuclei as well as hypothalamic area, amygdala, thalamus, and entorhinal cortex (Kalaitzakis et al., 2013). Rapid eye movement sleep behavior disorder in patients with PD was associated with disruptions of medullary and pontine circuits, which were vital in controlling sleep atonia in the period of rapid eye movement. In this study, we found that neurodegeneration indicated by the significant elevation of T-tau in CSF highly correlated with overall NPSs in patients with PD (Table 6). Thus, PD-NPSs might be related to the degeneration of neurons in specific brain regions and secondary disturbances of multiple neurotransmitters.

Interestingly, we simultaneously found that oxidative distress was reflected by the robust elevations of H_2_O_2_ and NO in CSF (Table 3); what about the correlation between oxidative distress and T-tau in PD-NPSs? A number of studies showed that oxidative distress was involved in neurodegeneration, which further aggravated oxidative distress, forming a vicious circle and promoting the occurrence and progression of PD (Dryanovski et al., 2013; Tapias et al., 2017). Different brain regions have different sensitivities to oxidative distress, among which, substantia nigra is one of the most sensitive areas indicated by the enhanced oxidative distress due to the great oxygen consumption and high levels of dopamine and its metabolites in dopaminergic neurons. However, no studies have focused on the relationship among oxidative distress, neurodegeneration, and PD-NPSs. In this study, the levels of H_2_O_2_ and NO significantly and positively correlated with the T-tau level in CSF from the PD-NPSs group (Table 7), which verified our speculation that oxidative distress was involved in neurodegeneration, and thereby aggravated PD-NPSs.

The hypothesis implied that reducing oxidative distress might be a plausible target for the intervention of PD-NPSs, which are called anti-oxidative therapy. Currently, monoamine oxidase B inhibitors (MAO-BIs), especially the new generation of MAO-BIs, have been used to improve motor symptoms of PD. In addition to the dopaminergic effect, it also has anti-oxidative action. However, oxidative scavenger and endogenous antioxidant supplements have not been widely used in the treatment of PD. Some clinical trials showed that antioxidants, such as vitamin E, coenzyme Q10, and creatine had neuroprotective effects, which were, however, limited and remained controversial (Beal et al., 2014; Jin et al., 2014). N-acetylcysteine and thymine quinone were effective in preclinical studies, but have not been tested in clinical trials yet (Martínez-Banaclocha, 2012; Ebrahimi et al., 2017). In conclusion, the therapeutic effects and side effects of the above drugs in patients with PD-NPSs are in urgent need of evidence-based research.

The limitation of this study is a lack of CSF samples from control subjects due to the difficulty in recruiting CSF from the normal elderly population. Hence, we searched the literature and compared the levels of neuroinflammatory factors in CSF between the PD-nNPSs group in our study and a normal control group in related literature. The comparisons showed that the levels of TNF-α, IL-1β, and IL-6 in CSF from the PD-nNPSs group in our study were significantly higher than those in normal control group reported by the literature, except that the IL-6 level in one study was higher than that in the PD-nNPSs group of our study. As neuroinflammation is the key pathogenesis of PD, it is reasonable that the levels of neuroinflammatory factors are higher in the PD-nNPSs group than normal control group. However, IL-6 level was higher in the normal control group of one study than the PD-nNPSs group in our study, which might be related to the different demographic variables, particularly, the age, or the kit used for the measurement of NO, etc. In the future, we will try our best to collect CSF samples and add neuroimaging information to further demonstrate the conclusion of this investigation.

## Conclusion

NPSs are prevalent in patients with PD, and patients with PD-NPSs show more advanced disease progression and severer motor symptoms than patients with PD-nNPSs; oxidative distress is characterized by the robust productions of free radicals (H_2_O_2_ and NO) and potential redistribution of anti-oxidative enzyme (tSOD) may closely correlate with neurodegeneration in NPSs-related brain regions, which may promote the occurrence and progression of PD-NPSs. Thus, in addition to symptomatic therapy by drug, antioxidant therapy may become an important and promising approach for PD-NPSs.

## Data availability statement

The raw data supporting the conclusions of this article will be made available by the authors, without undue reservation.

## Ethics statement

The studies involving human participants were reviewed and approved by Ethics Committee of Beijing Tiantan Hospital. The patients/participants provided their written informed consent to participate in this study.

## Author contributions

D-nL and WZ: designed the research. W-JZ, Y-nZ, PG, T-hL, H-yG, J-hL, M-yH, Wen-jZ, Wei-jZ, D-mL, and X-mW: performed research. D-nL and W-JZ: analyzed data. D-nL: wrote the paper. All authors read and approved the final manuscript.
